# Evaluating the rational use of antidepressant in older patients: a comprehensive analysis of its association with cognitive impairment

**DOI:** 10.3389/fpsyt.2025.1624989

**Published:** 2025-10-20

**Authors:** María Gil-Peinado, Juan Pardo, Mar García-Zamora, Gonzalo Miguel Adsuar-Meseguer, José Sendra-Lillo, Lucrecia Moreno

**Affiliations:** ^1^ Cathedra DeCo MICOF-CEU UCH, Valencia, Spain; ^2^ Muy Ilustre Colegio Oficial de Farmacéuticos de Valencia, Valencia, Spain; ^3^ Department of Mathematics, Physics and Technology. Universidad Cardenal Herrera-CEU, CEU Universities, Valencia, Spain; ^4^ Research Group in Alzheimer Disease. Instituto de Investigación Sanitaria La Fe, Valencia, Spain; ^5^ Department of Pharmacy, Universidad Cardenal Herrera-CEU, CEU Universities, Valencia, Spain

**Keywords:** cognitive impairment, depression, antidepressive agents, pharmacological therapy, rational use of medication

## Abstract

**Introduction:**

Dementia and Major Depressive Disorder (MDD) are on the rise globally, with depression frequently observed throughout the progression of dementia, potentially accelerating cognitive decline and diminishing quality of life. This study aims to explore the interplay between cognitive impairment (CI) and depression in patients undergoing antidepressant treatment, emphasizing drug-related problems (DRPs) and the Rational Use of Medicines (RUM).

**Materials and Methods:**

Over a 6-year period, this cross-sectional study in Valencia, Spain, analyzed data from 777 patients aged over 50 concerned about their cognitive health. Cognitive status was assessed using three neuropsychological tests: Memory Impairment Screening (MIS), Verbal Semantic Fluency (VSF), and Pfeiffer’s Short Portable Mental State Questionnaire (SPMSQ). Various clinical and demographic variables associated with dementia were also evaluated.

**Results:**

The study identified a higher prevalence of CI among patients at risk of depression (GDS5 positive) compared to those without a depression risk. Patients with depression risk also demonstrated lower cognitive reserve, higher levels of loneliness, and increased use of antidepressants – notably tricyclic antidepressants (TCAs) – which are linked to anticholinergic burden and potential CI.

**Conclusion:**

Despite their widespread use, antidepressants raise concerns regarding their efficacy and safety, particularly due to the risk of exacerbating CI. This study underscores the need for careful management of antidepressant therapy and suggests exploring alternatives such as vortioxetine, which may offer cognitive benefits. Enhanced interprofessional collaboration and regular cognitive evaluations are recommended to improve patient outcomes and ensure the rational use of antidepressants.

## 1 Introduction

According to the World Alzheimer Report 2023, more than 55 million people currently live with dementia worldwide (1). Additionally, the World Health Organization (WHO) estimates that this number will increase to 78 million by 2030 and to 139 million by 2050 (2). Concurrently, the prevalence of Major Depressive Disorder (MDD), also known as clinical depression, has been rising in recent years. Approximately 280 million people worldwide suffer from depression, which represents about 3.8% of the global population (3).

Alzheimer’s Disease (AD) is the most prevalent form of dementia, accounting for 60% to 80% of all major cognitive disorders (4). It is now recognized as a biological continuum that starts with preclinical AD—an early stage where symptoms are minimal or absent—and advances to its more severe form: dementia (5). This progression involves a gradual accumulation of pathophysiological changes over many years, eventually resulting in clinically apparent disease and a subsequent decline in cognitive and functional abilities. This decline occurs without distinct boundaries between clinical stages (6). AD is believed to begin up to 20 years before the onset of noticeable symptoms, highlighting a lengthy preclinical phase that may offer opportunities for early intervention, especially given the current lack of curative pharmacological treatments (7).

The NIA-AA (National Institute on Aging – Alzheimer’s Association) categorizes the progression of AD into six stages. These range from the asymptomatic presence of abnormal biomarkers (Stage 1) to severe dementia (Stage 6). Intermediate stages include the emergence of mild symptoms (Stage 2), Mild Cognitive Impairment (MCI) without significant functional loss (Stage 3), and advancing to mild, moderate, and severe dementia (Stages 4-6), with increasingly impaired independence. This staging system is especially valuable in clinical trials, as it facilitates the classification of patients according to the severity of their disease (4).

Dementia and depression are closely associated conditions. Depression is a commonly observed manifestation throughout the clinical progression of dementia, occurring both at the onset of cognitive decline and in later stages as the disease progresses (8, 9). The exact etiopathogenic relationship between both conditions remains unclear, but it is widely accepted that they share some underlying neurological basis (10).

The coexistence of depression with dementia has been linked to a faster progression of cognitive impairment (CI) and lower quality of life (11, 12). Most studies conclude that patients with depression tend to exhibit more pronounced CI compared to those who are not depressed (13). Some theories propose that depression in older adults may stem from a psychological response to perceived cognitive decline, thereby suggesting a potential association between the onset of depression and CI (14). Other studies suggest that late-life depression is a risk factor for dementia, potentially heightening the probability of transitioning from MCI to full-blown dementia (15). However, the question of whether depression arises because of the condition or precedes dementia as a prodromal symptom is still under investigation (16).

Shared impairments, such as memory loss, sleep disturbances, and reduced social functioning, are prevalent in both depression and dementia. Their association may be further explained by genetics or common pathophysiological pathways, including neurodegeneration, inflammation, vascular risk factors, and hypothalamic-pituitary-adrenal axis dysregulation. Regardless of the exact nature of this link, the comorbidity of depressive symptoms and dementia is well-established and must be considered in the care of patients with CI or dementia. Therefore, it is crucial to determine whether treating depression can improve cognitive functioning (16, 17).

The main objective of WHO policy brief is to support successful implementation of the third WHO Global Patient Safety Challenge, “Medication Without Harm”, and to advocate for prioritizing medication safety within healthcare systems (18). The use of antidepressants in neurological diseases is very common in daily clinical practice, primarily due to the close relationship between psychiatric comorbidities and neurological conditions (14). All antidepressants work slightly differently, targeting specific neurotransmitters to modulate mood and behavior. Classical antidepressants primarily increase levels of serotonin, norepinephrine, or both in the synapse. However, newer (atypical) antidepressants also increase dopamine levels, act as antagonists of dopamine D2 receptors, and serve as 5-HT2A antagonists and 5-HT1A agonists. Additionally, they may antagonize α2 receptors or utilize a novel multimodal mechanism of serotonin modulation and stimulation, such as seen with vortioxetine. Vortioxetine’s pharmacodynamic profile is unique, combining serotonin transporter blockade with a range of modulatory effects on serotonin receptors. The 5-HT1A agonist and 5-HT3 antagonist activities are considered crucial for reducing the typical latency of action seen with most antidepressants and for improving cognitive symptoms (19). Furthermore, vortioxetine functions as a 5-HT1B partial agonist, 5-HT1D antagonist, and 5-HT7 antagonist (**Table 1**).

**Table 1:** Classification of antidepressants authorized and marketed in Spain according to their pharmacological profile and anticholinergic burden.

Antidepressant		ATC Code	NA	5-HT	DA	Ach	α1	α2	5-HT1	5-HT2	H1	NA selectivity versus 5-HT	Anticholinergic burden
TCAs	Imipramine	N06AA02	++	++	+	++	++	+	+	+	++	NA	3
	Clomipramine	N06AA04	++	+++	+	+++	++	+	+	++	++	5-HT	3
	Amitriptyline	N06AA09	++	++	+	+++	+++	++	+	++	+++	NA	3
	Nortriptyline	N06AA10	+++	+	+	+	++	+	+	++	++	NA	3
	Doxepin	N06AA12	++	+	+	++	+++	+	+	++	+++	NA	3
	Maprotiline	N06AA21	+++	+	+	+	+++	+	0	+	+++	NA	2
SSRIs	Fluoxetine	N06AB03	+	+++	+	+	+	0	0	+	+	5-HT	1
	Citalopram	N06AB04	+	+++	+	0	+	0	0	+	+	5-HT	1
	Paroxetine	N06AB05	++	+++	+	++	+	+	0	0	0	5-HT	2
	Sertraline	N06AB06	+	+++	++	+	+	+	0	+	+	5-HT	1
	Fluvoxamine	N06AB08	+	+++	+	0	+	+	0	+	0	5-HT	1
	Escitalopram	N06AB10	0	+++	0	+	+	0	0	+	+	5-HT	1
SNRIs	Venlafaxine	N06AX16	+	++	+	0	0	0	0	0	0	5-HT	1
	Duloxetine	N06AX21	++	++	+	0	0	0	0	0	0	5-HT	0
	Agomelatine	N06AX22	0	0	0	0	0	0	0	++	0	5-HT	0
	Desvenlafaxine	N06AX23	++	+++	0	0	0	0	0	0	0	NA/5-HT	1
SNRI	Reboxetine	N06AX18	+++	+		0	+			+	+	NA	0
NDRI	Bupropion	N06AX12	+	0	++	0	+	0	0	0	+	NA	1
Others	Mianserin	N06AX03	+++	+	0	+	+++	+++	0	+++	+++	NA	0
	Trazodone	N06AX05	+	+	0	0	++	++	++	+++	+	5-HT	1
	Mirtazapine	N06AX11	+	+	0	+	+	++	0	++	+++	NA/5-HT	1
Multimodal	Vortioxetine	N06AX26	0	++	0	0	0	0	+	0	0	5-HT	0

(NA: noradrenaline reuptake blockade; 5-HT: serotonin reuptake blockade; DA: dopamine reuptake blockade; Ach: cholinergic receptors; α1: α1 receptors; α2: α2 receptors; 5-HT: serotonin receptors; H1: histamine receptors; 0: no effect; +: minimal effect; ++: moderate effect; +++: pronounced effect. Groups include tricyclics (TCA), selective serotonin re-uptake inhibitors (SSRI), serotonin and noradrenaline re-uptake inhibitors (SNRI), selective noradrenaline re-uptake inhibitors antidepressants (NSRI), noradrenaline and dopamine re-uptake inhibitors (NDRI), multimodal and others) Elaborated based on: Brunton L, Knollmann B, editors. Goodman and Gilman’s The Pharmacological Basis of Therapeutics. 14th ed. New York: McGraw-Hill Education; 2022.

The use of antidepressant medication has been implicated in the acceleration of CI symptoms, potentially through mechanisms such as increased anticholinergic burden and adverse vascular effects. These pharmacological impacts may contribute to an elevated risk of developing dementia (14, 20). Additionally, older adults may be more susceptible to these adverse effects due to age-related changes in pharmacokinetics and pharmacodynamics, including decreased acetylcholine-mediated transmission in the brain and increased permeability of the blood-brain barrier (21).

This underscores the need for more specific knowledge about which drugs would provide the maximum benefit for patients (22). However, this research includes challenges in accurately assessing and categorizing all present alterations, as well as the diversity of pharmacological treatments available.

The Rational Use of Medicines (RUM) is defined as the process through which “patients receive medications appropriate to their clinical needs, in doses that meet their own individual requirements, for an adequate period of time, and at the lowest cost to them and their community” (23). In accordance with Pharmaceutical Care Network of Europe (PCNE), a Drug-Related Problem (DRP) is defined as, “an event or circumstance involving drug therapy that actually or potentially interferes with desired health outcomes” (24). DRPs are classified based on where the failure occurs: need, safety, or effectiveness (25). Necessity refers to instances in which a medication is unnecessary, such as when a patient receives treatment without a valid clinical indication or when there is an unclear problem or complaint requiring further clarification before pharmacological intervention. Effectiveness pertains to problems related to the absence or potential absence of the desired therapeutic effect, which may arise from inappropriate drug selection, dosing, or adherence issues that hinder achieving optimal clinical outcomes. Finally, safety involves situations where the patient experiences or is at risk of experiencing adverse drug events, including side effects or toxicities, that compromise patient health (26).

Despite the risk of anticholinergic burden, antidepressants are prescribed to treat and prevent depression because they are considered safe, effective, and necessary. However, anticholinergic drugs are often non-selective, and their prolonged use can lead to severe adverse events such as CI (27).

Through this study, we aim to investigate the association between the risk of CI and depression in patients undergoing antidepressant treatment with potential DRP.

## 2 Materials and methods

### 2.1 Study design

This cross-sectional study was conducted in health centers, pharmacies, and various patients’ associations across the province of Valencia (Spain), utilizing simple random sampling.

The different clinical and demographic data were collected contemporaneously with the screenings carried out from 2018 to 2024 through patient interviews. These data are part of the Cathedra DeCo project.

The study of human subjects has ethical implications. This study was reviewed and approved by the Ethical Committee for Clinical Research with Medications of the Arnau de Vilanova Health Department (MOR-ROY-2018–013, date of approval: 18 July 2018). All participants signed informed consent to participate in the study.

### 2.2 Cognitive status

To detect those patients with possible CI, three neuropsychological tests were performed, following the recommendations of the local government through the Conselleria de Sanitat de la Comunitat Valenciana (28). Thus, patients were assessed with the following tests: Memory Impairment Screening (MIS), Verbal Semantic Fluency (VSF) and Pfeiffer’s Short Portable Mental State Questionnaire (SPMSQ). The MIS was validated in Spanish by Böhm et al., with a maximum score of 8 and a cut-off point of 4 or less. In the initial phase, participants are required to read aloud four related words from different categories. Following an unstructured distraction period, a free recall test is conducted, with semantic cues provided for words or categories that the participant cannot recall. Freely recalled items are awarded 2 points, while those recalled with a cue receive 1 point, resulting in a scoring range from 0 to 8 (29). The VSF was validated in Spanish by López Pérez-Díaz et al., with a cut-off point of 10. This test measures the number of items within a category that a subject can recall in one minute (30). The SPMSQ was validated in Spanish by Martínez de la Iglesia et al, with a maximum score of 10 and a cut-off point of 3 errors (4 errors for illiterate individuals). This test evaluates various aspects of intellectual functioning, including short-term memory, long-term memory, current event information, orientation, and the ability to perform serial mathematical tasks (31). The sensitivity, specificity and test duration of the above are shown in **Table 2**. The complementary use of the three tests aims to increase the likelihood of detecting cases of CI, since, in some instances, the combination of multiple questionnaires may represent the most appropriate strategy for a comprehensive evaluation (28). A positive screening result is considered when any of the tests exceeds the validated clinical threshold, in line with our objective of promoting early identification.

**Table 2:** Sensitivity, specificity, and time duration of the short test used in cognitive impairment detection.

Screening test	Sensibility	Specificity	Duration (minutes)	Cut-off points	Test score (mean ± sd)
MIS	0,74	0,96	2	≤ 4	6.66 ± 1.84
SPMSQ	0,85	0,79	3	≥ 3	0.99 ± 1.22
VSF	0,74	0,80	1	10	18.53 ± 7.09

(MIS, Memory Impairment Screening; SPMSPQ, Pfeiffer’s Short Portable Mental State Questionnaire; VSF, Verbal Semantic Fluency).

Patients with at least one positive cognitive test were classified as individuals with CI and those who did not fail any test as patients without CI. Consequently, subjects with a score compatible with the presence of CI in any of the three tests were referred to Primary Care for medical diagnosis.

### 2.3 Variables

In addition to the different neuropsychological tests, different clinical and demographic variables were collected, including age, sex, marital status, study level, hearing loss, group activities, number of friends seen in the last week, and subjective memory complaint (SMC).

We also included the Cognitive Reserve Questionnaire (CRQ) (32), where values less than or equal to 6 indicate low cognitive reserve, values between 7 and 9 indicate medium/low cognitive reserve, values between 10 and 14 indicate medium/high cognitive reserve, and values greater than or equal to 15 indicate high cognitive reserve; the Sense Of Coherence (SOC) (33); the Purpose In Life (PIL) (34); the Engaged Living Scale (ELS) (35); a Brief Resilient Coping Scale (BRCS) (36), where values below 13 indicate low resilience, values between 13 and 17 indicate intermediate resilience, and values above 17 indicate high resilience; the Loneliness Scale (UCLA) (37), where values higher or equal to 6 are associated with loneliness; and the Yesavage Scale For Geriatric Depression (GDS5) (38), where values greater than or equal to 2 are associated with a risk of depression. Prior validation studies in older Spanish populations demonstrated that this threshold balances sensitivity and specificity effectively for screening purposes (39). Finally, antidepressant drug (N05 and N06 ATC codes) prescription was also recorded. Information on current medication prescriptions was extracted from patients’ electronic health records at the time of cognitive assessment. The list of antidepressants considered, along with their dosages and frequencies, is provided in Appendix I.

### 2.4 Study subjects

The initial population consisted of 1,086 patients over the age of 50 who were concerned about their cognitive health and interested in undergoing screening for CI, provided they met the selection criteria. These criteria included: age ≥50 years, presence of subjective memory complaints, and provision of informed consent. Patients were excluded if they had a diagnosis of Alzheimer’s disease (AD) or other dementias, mental illnesses, or significant sensory deficits. A diagnosis of depression and/or the use of antidepressants at the time of the surveys was not part of the inclusion or exclusion criteria for this study.

Participants were recruited through three main pathways (1): the service was offered by community pharmacists directly to patients at the pharmacy (2); referral from primary care physicians; or (3) patients proactively sought participation after learning about the project. Data collection was carried out through structured interviews with the patient, conducted at the community pharmacy, the primary care center, or a patient association facility.

**Figure 1:**
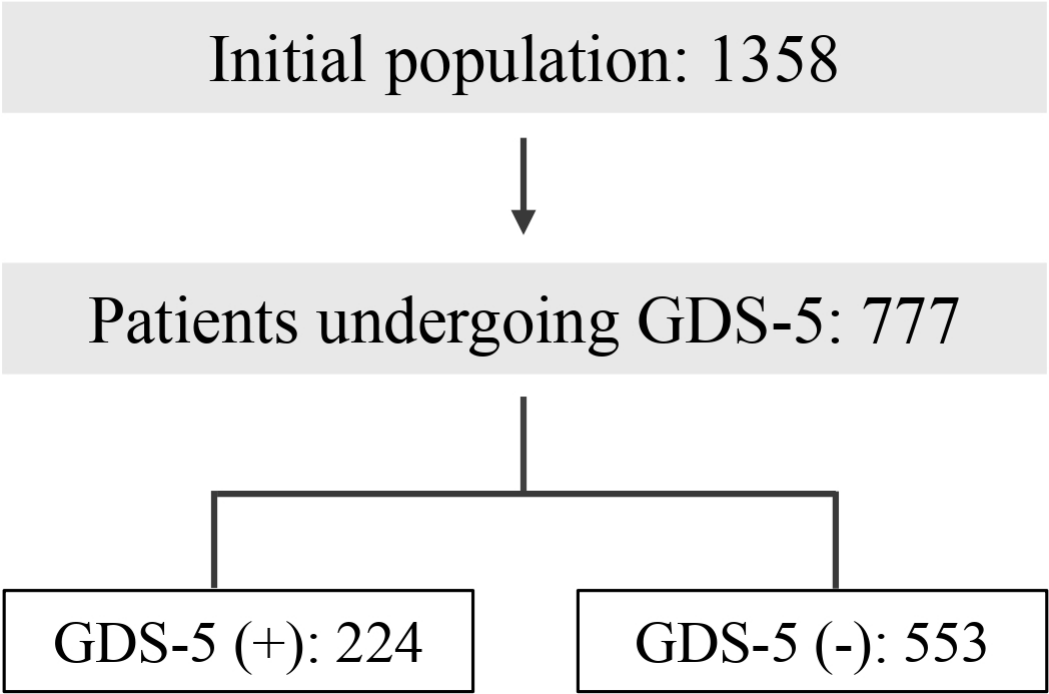
Study population (GDS5: 5 Point Geriatric Depression Scale).

As shown in **Figure 1**, for this specific study, only the 777 patients with available GDS5 data were included. To calculate whether we had sufficient statistical power, we used the G*Power program [REF] to determine the minimum sample size for the study, comparing two independent groups and assuming a medium effect size with a significance level (Alpha) of 0.05. Finally, the statistical power achieved is about 0.99 (40).

### 2.5 Statistical analysis

A descriptive analysis was carried out to compare the different variables between patients with GDS5 risk and patients without GDS5 risk. Categorical variables were compared by the Pearson’s Chi-squared test or the nonparametric alternative through Fisher’s exact test when appropriate. They were depicted by percentages. Numerical variables were represented through mean and standard deviation and compared with two-sample t-tests after testing compliance with the assumptions of normality (Shapiro-Wilk test) and homogeneity of variance (Levene’s test). An equivalent non-parametric alternative to the t-test was employed if such assumptions were not met. In that sense, the Wilcoxon rank-sum method was used. All tests were two-tailed, and a p-value < 0.05 indicated statistical significance. In Appendix II, a logistic regression model adjusted by age and sex has been calculated to reinforce the discoveries of the statistical tests. Moreover, this study is an exploratory rather than a confirmatory study that aims to discover new working hypotheses on the relationship between antidepressant treatment and dementia. Then, we did not adjust p-values for multiple comparisons given the descriptive nature of this epidemiologic study, in line with existing recommendations (41).

Data analyses were performed using R (version 4.3.1) with R studio (version 2023.12.0.369) (42).

## 3 Results

Among the 777 patients who underwent the GDS5 test, 224 obtained a score within the range indicating a risk of depression, while 553 did not.

The distribution of scores for each cognitive test is summarized in **Table 2**. The average MIS score was (6.66 ± 1.84), with 113 (14.54%) participants scoring below the cut-off (≤4). On the SPMSQ, participants had a median of 0.99 ± 1.22, with 85 (10.94%) exceeding the cut-off for CI. The VSF test showed a median score (18.53 ± 7.09), and 48 individuals (6.18%) scored below the threshold of 10 words. These results show variability in cognitive performance among the study population (**Table 2**).

### 3.1 Dementia-related factors

**Figure 2:**
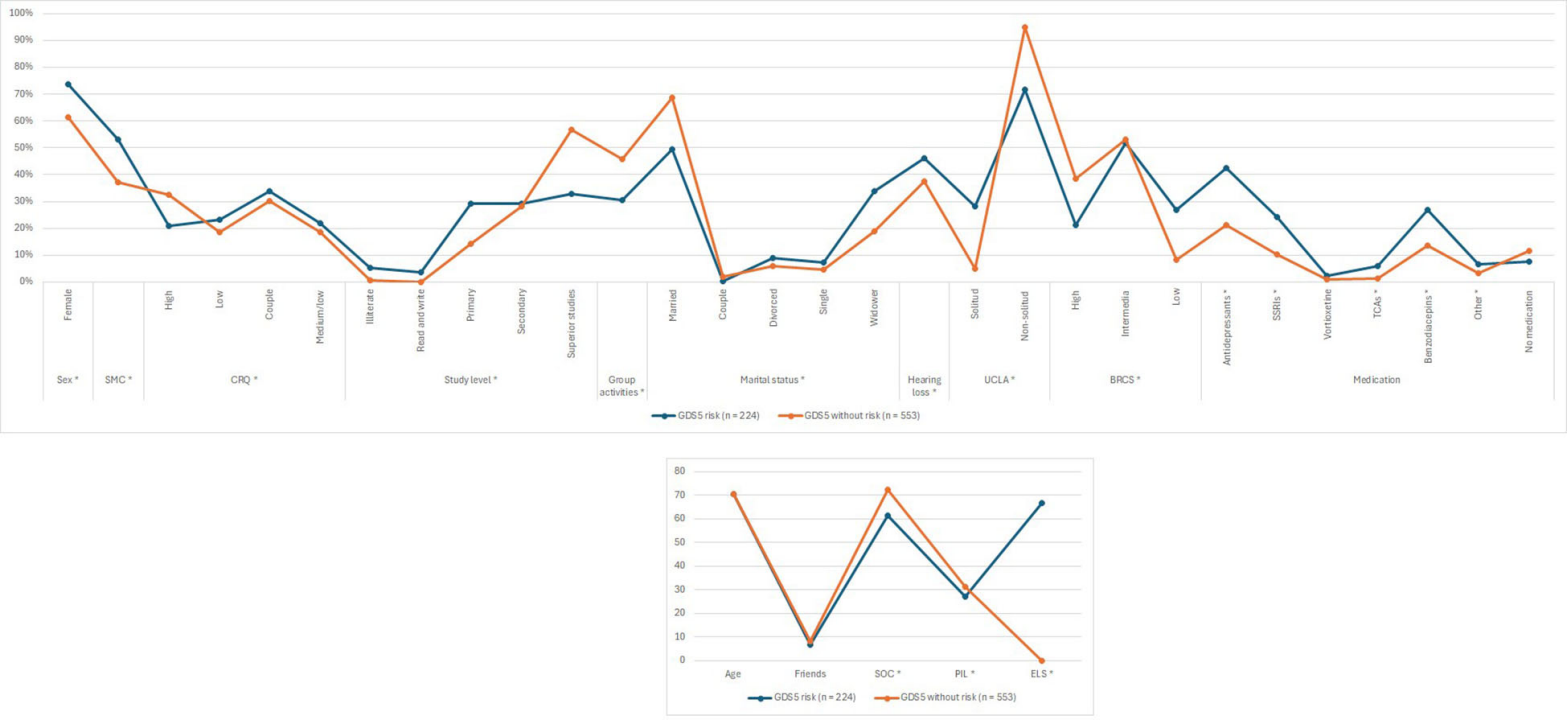
Association of dementia-related factors with depression risk estimated by the GDS5 (GDS5, 5 Point Geriatric Depression Scale; CI, Cognitive Impairment; SMC, Subjective Memory Complaint; CRQ, Cognitive Reserve Questionnaire; SOC, Sense of Coherence; PIL, Purpose in Life; ELS, Engaged Living Scale; BRCS, Brief Resilience Coping Scale; UCLA, University of California, Los Angeles Loneliness Scale; SSRIs, Serotonin Reuptake Inhibitors; TCAs, Tricyclic Antidepressants) The variables marked with an asterisk (*) are statistically significant.

As shown in **Table 3** and **Figure 2**, the percentage of patients with CI in the group of GDS5 risk was significantly higher than in patients without GDS5 risk [n = 59 (26.46%), n = 102 (18.61%); p-value = 0.0151]. Additionally, individuals with CI had higher odds of having GDS5 risk [ORs (CI 95%): 1.64 (1.12, 2.39); p-value = 0.0098] (LR model in Appendix II).

**Table 3:** Association of dementia-related factors with depression risk estimated by the GDS5.

Dementia related factors		GDS5 risk (n = 224)	GDS5 without risk (n = 553)	P-value
Age, mean(std) a		70.45 (12.49)	70.28 (11.03)	0.8516
Sex (Female) n(%) b		165 (73.66)	340 (61.48)	0.0013 *
SMC, n(%) b		119 (53.12)	206 (37.32)	0.0001 *
CI, n(%) b		59 (26.46)	102 (18.61)	0.0151 *
MIS	Mean(std) a	6.53 (1.80)	6.71 (1.85)	0.2161
	No risk, n(%) b	193 (86.16)	468 (84.63)	0.6483
	Risk, n(%) b	30 (13.39)	83 (15.01)	
SPMSQ	Mean(std) a	1.21 (1.35)	0.90 (1.15)	0.0013 *
	No risk, n(%) b	192 (85.71)	500 (90.42)	0.0759
	Risk, n(%) b	32 (14.29)	53 (9.58)	
VSF	Mean(std) a	16.84 (6.84)	19.21 (7.08)	<0.0001 *
	No risk, n(%) b	201 (89.73)	522 (94.39)	0.0123 *
	Risk, n(%) b	22 (9.82)	26 (4.70)	
CRQ b	High, n(%)	9 (20.97)	171 (32.51)	0.0292 *
	Low, n(%)	43 (23.12)	98 (18.63)	
	Medium/high, n(%)	63 (33.87)	159 (30.23)	
	Medium/low, n(%)	41 (22.04)	98 (18.63)	
Study level c	Illiterate, n(%)	3 (5.45)	1 (0.75)	0.0009 *
	Read and write, n(%)	2 (3.64)	0 (0.00)	
	Primary, n(%)	16 (29.09)	19 (14.18)	
	Secondary, n(%)	16 (29.09)	38 (28.363)	
	Superior studies, n(%)	18 (32.73)	76 (56.72)	
Group activities, n(%) b		57 (30.48)	241 (45.9)	0.0002 *
Marital status c	Married, n(%)	111 (49.55)	377 (68.55)	0.0001 *
	Couple, n(%)	1 (0.45)	11 (2.00)	
	Divorced, n(%)	20 (8.93)	33 (6.00)	
	Single, n(%)	16 (7.14)	25 (4.55)	
	Widower, n(%)	76 (33.93)	104 (18.91)	
Hearing loss (yes), n(%) b		n = 103 (45.98)	n = 208 (37.61)	0.0310 *
Friends, mean(std) a		6.70 (7.20)	8.20 (8.30)	0.3897
SOC, mean(std) d		61.20 (12.80)	72.30 (10.40)	<0.0001 *
PIL, mean(std) a		27.10 (6.10)	31.10 (4.60)	<0.0001 *
ELS, mean(std) d		66.50 (15.80)	77.30 (13.10)	<0.0001 *
UCLA Loneliness Scale b	Solitud, n(%)	43 (28.29)	17 (4.93)	<0.0001 *
	Non-solitud, n(%)	109 (71.71)	328 (95.07)	
BRCS b	High, n(%)	40 (21.39)	203 (38.59)	<0.0001 *
	Intermedia, n(%)	97 (51.87)	279 (53.04)	
	Low, n(%)	50 (26.74)	44 (8.37)	
Antidepressant medication	Antidepressants, n(%) b	95 (42.41)	118 (21.34)	<0.0001 *
	SSRIs, n(%) b	54 (24.11)	56 (10.13)	<0.0001 *
	Vortioxetine, n(%) c	5 (2.23)	5 (0.90)	0.1620
	TCAs, n(%) b	13 (5.80)	8 (1.45)	0.0007 *
	Benzodiacepins, n(%) b	60 (26.79)	75 (13.56)	<0.0001 *
	Other, n(%) b	15 (6.70)	18 (3.25)	0.0312 *
	No medication, n(%) a	17 (7.59)	64 (11.57)	0.0997
Anticholinergic burden a	Burden, mean(std)	1.6 (1.9)	0.8 (1.3)	<0.0001 *
	Antidepressants, mean(std)	0.9 (1.3)	0.4 (0.9)	<0.0001 *
	Other drugs, mean(std)	0.7 (1.2)	0.5 (0.9)	0.0175 *

(GDS5: 5 Point Geriatric Depression Scale; CI: Cognitive Impairment; SMC: Subjective Memory Complaint; CRQ: Cognitive Reserve Questionnaire; SOC: Sense of Coherence; PIL: Purpose in Life; ELS: Engaged Living Scale; BRCS: Brief Resilience Coping Scale; UCLA: University of California, Los Angeles Loneliness Scale; SSRIs: Serotonin Reuptake Inhibitors; TCAs: Tricyclic Antidepressants) The variables marked with an asterisk (*) are statistically significant. a Wilcoxon rank sum test (p-value < 0.05). b Chi-squared test (p-value < 0.05). c Fisher’s Exact Test (p-value < 0.05). d Two Sample t-test (p-value < 0.05).

Most patients with GDS5 risk were female [n = 165 (73.66%), n = 340 (61.48%); p-value = 0.0013]. Similarly, there were significantly more individuals with SMC in the GDS5 risk group than in the non-risk group [n = 119 (53.12%), n = 206 (37.32%); p-value = 0.0001]. Consistently, results presented in Appendix II show that participants with SMC had significantly higher odds of having GDS5(+) [Ors (CI 95%): 1.84 (1.34, 2.53); p-value = 0.0002]. In addition, participants with GDS5 risk had significantly higher punctuation in SPMSQ [1.21 (1.35), 0.90 (1.15); p-value = 0.0013] and lower punctuation in VSF [16.84 (6.84), 19.21 (7.08); p-value < 0.0001].

When talking about cognitive reserve, there were more patients with CRQ high marks in the group without risk [n = 39 (20.97%), n = 171 (32.51%); p-value = 0.0292]. Nevertheless, the percentage of patients with low [n = 43 (23.12%), n = 98 (18.63%); p-value = 0.0292], medium/low [n = 41 (22.04%), n = 98 (18.63%); p-value = 0.0292] and medium/high [n = 63 (33.87%), n = 159 (30.23%); p-value = 0.0292] marks was higher in the GDS5 risk group. Furthermore, there were significantly more patients with studies such as primary [n = 16 (29.09%), n = 19 (14.18%); p-value = 0.0009], secondary [n = 16 (29.09%), n = 38 (28.36%); p-value = 0.0009], or superior [n = 18 (32.73%), n = 76 (56.72%); p-value = 0.0009] in the group without risk than in the group with risk. Likewise, there were more patients participating in group activities in the group without risk [n = 57 (30.48%), n = 241 (45.90%); p-value = 0.0002].

Regarding the marital status, there were more patients married [n = 111 (49.55%), n = 377 (68.55%); p-value = 0.0001] and as a couple [n = 1 (0.45%), n = 11 (2.00%); p-value = 0.0001] in the group without risk of depression. In contrast, there were more patients divorced [n = 20 (8.93%), n = 33 (6.00%); p-value = 0.0001], single [n = 16 (7.14%), n = 25 (4.55%); p-value = 0.0001] and widowed [n = 76 (33.93%), n = 104 (18.91%); p-value = 0.0001] in the group with risk of depression.

The percentage of patients with GDS5 risk with hearing loss was significantly higher than patients without risk [n = 103 (45.98%), n = 208 (37.61%); p-value = 0.0310]. Moreover, individuals with GDS5 risk were significantly lonelier than those without risk. This is shown in the UCLA test [n = 43 (28.29%), n = 17 (4.93%); p-value < 0.0001], in the number of friends [6.70 (7.20), 8.20 (8.30); p-value = 0.3897] and in the percentage of patients that practice any group activity [n = 57 (30.48%), n = 241 (45.90%); p-value = 0.0002].

Resilience (BRCS), sense of coherence (SOC) [61.20 (12.80), 72.30 (10.40); p-value < 0.0001], purpose in life (PIL) [27.10 (6.10), 31.10 (4.60); p-value < 0.0001] and the engaged living scale (ELS) [66.50 (15.80), 77.30 (13.10); p-value < 0.0001] were also significantly higher in patients without risk of depression than in those with risk.

Finally, the percentage of individuals taking antidepressants [n = 95 (42.41%), n = 118 (21.34%); p-value < 0.0001] was higher in the GDS5 risk group. Specifically, SSRI [n = 54 (24.11%), n = 56 (10.13%); p-value < 0.0001], TCAs [n = 13 (5.80%), n = 8 (1.45%); p-value = 0.0007] and other antidepressants [n = 15 (6.70%), n = 18 (3.25%); p-value = 0.0312] (**Table 3**).

### 3.2 Rational use of antidepressant treatment

#### 3.2.1 Necessity of antidepressant treatment

This section analyzes the necessity of antidepressant treatment in the group of patients classified as high-risk according to the GDS5 scale. The results show that a significantly higher proportion of patients (57.59%, total: n = 129) were not receiving antidepressant treatment compared to those who were (42.41%, total: n = 95), with a statistically significant difference [X^2^ (1) = 10.30; p-value = 0.0001]. However, as shown in Appendix II, individuals using antidepressants had higher likelihood of having GDS5(+).

#### 3.2.2 Efficacy of antidepressant treatment

Regarding the efficacy of antidepressant treatment, in the group of patients classified as without risk according to the GDS5 scale, the results show that a significant majority of patients (78.66%, total: n = 435) were not receiving antidepressant treatment, compared to a smaller percentage who were (21.34%, total n: = 118), with a statistically significant difference [X^2^ (1) = 363.06; p-value < 0.0001].

#### 3.2.3 Safety of antidepressant treatment

Concerning the safety of antidepressant treatment, as shown in **Table 4**, within the GDS5-risk group, significantly more patients with CI (34.04%, total: n = 32) and SMC (65.26%, total: n = 62) were taking antidepressants than those who were not (CI: 20.93%, total: n = 27 and, SMC: 44.19%, total: n = 57) [CI: X^2^ (1) = 9.64; p-value = 0.0284 and SMC: X^2^ (1) = 20.02; p-value = 0.0018]. Similarly, within the GDS5-without-risk group, a significantly higher number of patients with SMC were also receiving antidepressant treatment (51.69%, total: n = 61). However, no statistically significant differences were observed regarding CI in this group.

**Table 4:** Association of antidepressants usage with depression risk estimated by the GDS5.

GDS5 (+)			
	Non-antidepressants	Antidepressants	P-value ^a^
CI, n(%)	n = 27 (20.93%)	n = 32 (34.04%)	0.0284 *
SMC, n(%)	n = 57 (44.19%)	n = 62 (65.26%)	0.0018 *

GDS5 (–)			
	Non-antidepressants	Antidepressants	P-value ^a^
CI, n(%)	n = 76 (17.63%)	n = 26 (22.22%)	0.2581
SMC, n(%)	n = 145 (33.41%)	n = 61 (51.69%)	0.0003 *

(GDS5: 5 Point Geriatric Depression Scale; CI: cognitive impairment; SMC: subjective memory complaint) The variables marked with an asterisk (*) are statistically significant. ^a^ Chi-squared test (p-value < 0.05).

**Figure 3:**
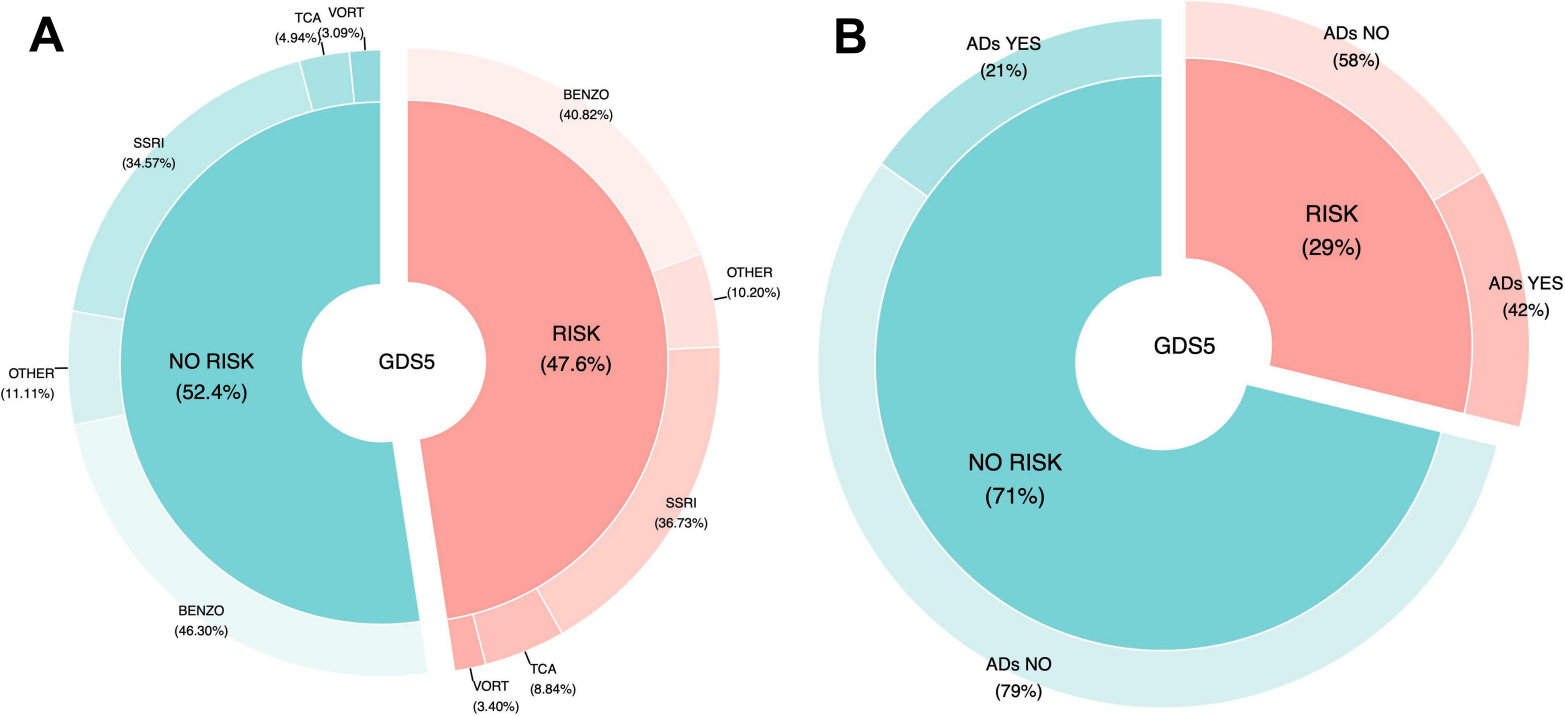
Association of antidepressants usage with depression risk estimated by the GDS5. **(A)** Comparison between participants using any antidepressant and those not using them, within the GDS-5 “risk” and “no-risk” groups. **(B)** Comparison among specific antidepressant classes in participants with and without depression risk according to the GDS-5. (GDS5, 5 Point Geriatric Depression Scale; ADs, Antidepressants; SSRIs, Serotonin Reuptake Inhibitors; TCAs, Tricyclic Antidepressants; VORT, Vortioxetine; BENZO, Benzodiazepines).

Furthermore, **Figure 3A** illustrates the estimated risk of depression, as measured by the GDS5, in relation to overall antidepressant consumption. In contrast, **Figure 3B** shows the risk of depression stratified by the type of antidepressant used. The data indicate that antidepressant treatment is associated with 42% ineffectiveness, 58% necessity, and 21% efficacy. Moreover, TCAs are consumed twice as frequently when the GDS5 result is positive compared to when it is negative.

## 4 Discussion

This study’s primary contribution is the examination of the association between depression and cognitive status, and how this relationship relates to the use of antidepressant treatment. In this cross-sectional study, conducted with a sample of 777 patients, we investigated the necessity, effectiveness, and safety of antidepressant treatment, as well as its association with cognitive status.

Despite its undefined etiology, SMC should not be considered a trivial symptom in the elderly population, as it may indicate current alterations in mood or cognition and predict the future onset of dementia (43). SMC has been associated with depressive symptoms in older adults with MCI, making early detection and management of these symptoms highly important (44). This is in line with our results, as CI and SMC were associated with depression risk, according to GDS5.

As expected, our study found that a lower cognitive reserve (measured by the CRQ, low levels of education, or lack of participation in group activities) was associated with an increased risk of depression. A previous study concluded that higher cognitive and brain reserve was associated with a reduced risk of experiencing depressive episodes in old age. However, this protective effect was lessened when individuals with clinically relevant depressive symptoms at the start of the study were excluded, indicating that the advantage of a greater cognitive and brain reserve in preventing depressive episodes in old age partly depends on the presence of existing depressive symptoms (45). Additionally, it has been proposed that while high cognitive reserve generally provides cognitive protection, depression may undermine this advantage, leading to greater cognitive difficulties. This underscores the complex interplay between cognitive reserve, mental health, and cognition (46).

We also observed an association between the risk of depression and loneliness (interpreted from the UCLA scale, divorced marital status and hearing loss). Although being divorced does not necessarily imply social isolation, it has been proposed that people who have a partner and live with someone may be more cognitively and socially active (15). On the other hand, multiple studies report associations between hearing loss, depression, and brain changes in middle-aged and older adults. While the direct relationship between depression arising from hearing loss and cognitive performance is still being investigated, the cumulative findings from current studies support the hypothesis that depression acts as a mediator between these factors (47). Meaning in Life (MiL) (according to SOC, PIL and ELS) and resilience (according to BRCS) were also associated with the risk of depression. Studies have shown that MiL may influence several risk factors for cognitive decline (48), as individuals who report higher levels of meaning engage in more physical activity and are less likely to have diabetes or high blood pressure, both of which are risk factors for dementia (49). Additionally, emerging research strongly supports the idea that a sense of purpose in life is linked to favorable cognitive outcomes in older adults, including enhanced cognitive performance and resilience against dementia-related neuropathology (50). Moreover, a higher sense of purpose in life appeared to mitigate some of the negative effects of depressive symptoms on memory performance (51).

According to the PCNE and the WHO, pharmaceutical care is based on the concept of the responsible use of medicines, which involves optimizing their effectiveness, efficiency, and safety (52). Regarding the rational use of antidepressant treatment in our study sample, the association between an increased risk of depression and lower antidepressant use may highlight the need for initiating treatment in certain cases. Conversely, the observed link between a higher risk of depression and increased antidepressant use suggests a potential need to reassess the current therapeutic strategies. Additionally, the observed association between lower depression risk and reduced use of antidepressants may reflect that individuals with fewer depressive symptoms do not require pharmacological treatment. This analysis highlights a potential deficiency in access to appropriate treatment or a lack of early diagnosis, which is crucial for early pharmaceutical intervention. These findings emphasize the importance of closely follow up in the management of antidepressant therapy to optimize treatment outcomes (53, 54).

Several studies have examined the risk of cognitive decline with anticholinergic medication use in elderly patients, consistently finding an association with poor cognitive performance in various settings (55–58). They recognize the critical need to optimize its use among elderly adults and agree that it should be discouraged when suitable alternatives are available. Our results indicated that the consumption of TCAs doubled when the GDS5 score was positive. This finding is particularly notable given that TCAs have a high anticholinergic burden (59), as shown in **Table 1**.

Although a recent study showed that elderly users of SSRIs and other antidepressants have a higher risk of developing dementia than elderly users of TCAs (60), a previous meta-analysis indicated that the use of antidepressants, particularly TCAs and SSRIs, is associated with an increased risk of CI in older adults, regardless of the presence of dementia (61). Additionally, TCAs and Monoamine Oxidase Inhibitors (MAOIs) may have a higher association with executive function impairment compared to other classes of antidepressants, such as Serotonin-Norepinephrine Reuptake Inhibitors (SNRIs) (62). For instance, TCAs like nortriptyline have been linked to lower baseline cognitive performance in areas such as verbal functions, visual memory, and psychomotor speed. These medications can exacerbate cognitive deficits due to their anticholinergic effects, which interfere with acetylcholine neurotransmission, a key factor for memory and learning. Furthermore, TCAs carry a higher risk of cardiovascular side effects and overdose toxicity, making them less advisable for older adults (63, 64).

While the anticholinergic action of TCAs is the primary factor contributing to CI, significantly affecting cholinergic receptors (57), their impact on other receptors, such as histaminergic and adrenergic, also plays a role in their overall side effect profile, though to a lesser extent. These combined effects likely account for the heightened risk of CI associated with the use of TCAs (65) TCAs are known to cause CI through several mechanisms, including their interaction with histaminergic and adrenergic receptors. Specifically, TCAs block H1 histamine receptors, leading to sedation and drowsiness, which can impair attention and memory. This prolonged sedation interferes with cognitive functions, especially in tasks requiring focus and memory retention. Additionally, TCAs block α1-adrenergic receptors, which contributes to orthostatic hypotension and dizziness, further complicating cognitive processing and concentration abilities. This combination of receptor interactions can lead to both cognitive and mood disturbances, particularly affecting elderly or sensitive patients (60).

For those patients diagnosed with depression who remain untreated or do not improve with their current antidepressants, alternatives should be considered. Vortioxetine (66), a multimodal antidepressant with potential procognitive effects, could enhance the safety of antidepressant treatment (67), quality of life and health outcomes (68) related to depression. Unlike other antidepressants, Vortioxetine (69) acts as a modulator of multiple serotonergic receptors, including serotonergic receptors (5-HT1A, 5-HT1B, 5-HT1D, 5-HT3, and 5-HT7), as well as a serotonin reuptake inhibitor and 5-HT1A receptor partial agonist. These mechanisms uniquely modulate serotonergic neurotransmission, offering broader effects than simply elevating serotonin levels.

### 4.1 Strengths and Limitations

Data collection was based on a comprehensive face-to-face interview lasting approximately 90 minutes, ensuring the acquisition of detailed and reliable information on dietary intake and lifestyle. Cognitive function was assessed using three complementary tests (MIS, SPMSQ, and SVF), each capturing different cognitive domains with varying degrees of sensitivity and specificity, thereby allowing for a broader and more nuanced evaluation of cognitive performance. Integrating all three assessments strengthens the ability to correctly identify individuals with CI, ultimately leading to greater diagnostic precision.

Our study has certain limitations, primarily due to the low percentage of patients diagnosed with depression currently receiving treatment with vortioxetine (3.40%). During the period from 2020 to 2024, vortioxetine has not been widely adopted as a primary treatment option in clinical practice, especially compared to other medication groups such as SSRIs. Furthermore, an inherent limitation of cross-sectional studies is their inability to establish causal inference. However, they enable the identification of associations that can generate new hypothesis for future research.

Another limitation of the present study is the use of the GDS-5, a self-reported screening instrument, rather than a clinician-rated diagnostic tool for depression. While the GDS-5 is practical and validated for use in large-scale studies, it may not fully capture clinically diagnosed depression and can be subject to self-reporting bias.

Although CI risk was estimated using validated screening questionnaires, they are widely employed in Primary Care settings. This approach aligns with routine clinical practice for early detection. Importantly, participants who screened positive were subsequently referred for comprehensive clinical evaluation and diagnostic confirmation. This two-step process ensures both practical applicability in primary care and reliability in identifying true cases of CI.

Finally, while the sample size was sufficient to detect small-to-moderate associations, the exploratory nature of the study calls for confirmation through longitudinal or interventional research.

## 5 Conclusion

This study underscores the importance of cognitive assessments and regular monitoring in patients undergoing antidepressant treatment, particularly in relation to the risk of CI and its association with depression. Strengthening interprofessional collaboration, especially between pharmacists, primary care providers, and neurology specialists, can significantly improve the safety, appropriateness, and effectiveness of antidepressant therapy. Such collaboration allows for the identification of patients receiving antidepressant treatment from community pharmacies who show limited improvement, enabling timely referrals to primary care or neurology units. This approach is crucial for addressing early CI, its link with depression, and the potential side effects associated with anticholinergic burden.

Additionally, further research is needed to explore the cognitive effects of vortioxetine. If its cognitive benefits are confirmed, updating prescription guidelines would be essential to optimize treatment outcomes for patients at risk of CI.

## Data Availability

The raw data supporting the conclusions of this article will be made available by the authors, without undue reservation.
